# Identification of potential genomic regions and candidate genes for egg albumen quality by a genome-wide association study

**DOI:** 10.5194/aab-62-113-2019

**Published:** 2019-03-25

**Authors:** Liang Qu, Manman Shen, Jun Guo, Xingguo Wang, Taocun Dou, Yuping Hu, Yongfeng Li, Meng Ma, Kehua Wang, Honglin Liu

**Affiliations:** 1 College of Animal Science & Technology, Nanjing Agricultural University, Nanjing, China; 2 Jiangsu Institute of Poultry Science, Chinese Academy of Agricultural Science, Yangzhou, China; 3 College of Animal Science & Technology, Yangzhou University, Yangzhou, China

## Abstract

Albumen quality is a leading economic trait in the chicken industry. Major studies have paid attention to genetic architecture underlying albumen quality. However, the putative quantitative trait locus (QTL) for this trait is still unclear. In this genome-wide association study, we used an $F_{2}$ resource population to study longitudinal albumen quality. Seven single-nucleotide polymorphism (SNP) loci were found to be significantly ($p<8.43\times 10^{-7}$) related to albumen quality by univariate analysis, while 11 SNPs were significantly ($p<8.43\times 10^{-7}$) associated with albumen quality by multivariate analysis. A QTL on GGA4 had a pervasive function on albumen quality, including a SNP at the missense of *NCAPG*, and a SNP at the intergenic region of *FGFPB1*. It was further found that the putative QTLs at GGA1, GGA2, and GGA7 had the strongest effects on albumen height (AH) at 32 weeks, Haugh units (HU) at 44 weeks, and AH at 55 weeks. Moreover, novel SNPs on GGA5 and GGA3 were associated with AH and HU at 32, 44, and 48 weeks of age. These results confirmed the regions for egg weight that were detected in a previous study and were similar with QTL for albumen quality. These results showed that GGA4 had the strongest effect on albumen quality. Only a few significant loci were detected for most characteristics probably reflecting the attributes of a pleiotropic gene and a minor-polygene in quantitative traits.

## 1 Introduction

Chicken eggs are one of the world's perfect table foods. Egg quality comprises external and internal quality, and can be defined by the eggshell, albumen, and yolk quality, and special emphasis on the importance of albumen quality, which is the major component (accounting for ∼60 %) of the whole egg (Campbell et al., 2003). From an exterior view, good albumen quality should keep the yolk in the center of the egg (Li-Chan and Kim, 2008) and is a very important parameter to indicate egg freshness. According to the interior structures of an egg, albumen contains many functionally important proteins and is a good protein food with complete amino acid composition (Abeyrathne et al., 2013). The thicker the albumen height, the grater the foaming, emulsifying, and gelling properties, and the proteome composition would be better, which is meaningful in the food industry (Sun et al., 2017). Thus, improving the egg albumen quality is the current focus of breeding purposes. At present, the most widely used criterion of albumen quality are the albumen height (AH) and Haugh unit (HU) (Haugh, 1937).

Egg albumen quality is affected by several factors including strain or breed, age, nutrition, storage, and disease (Roberts, 2004). The most important factor is the strain or breed, and studies have shown that different strains of hen vary significantly in albumen quality. The heritability estimates of AH were reported as moderate from 0.29 to 0.51 (Honkatukia et al., 2013), so albumen quality could be improved by genetic selection (Scott and Silversides, 2000).

Albumen quality decreases with the aging process, and previous reports focused on the genetics of albumen quality and are based on one time point, especially on week 40. Many of the quantitative trait loci (QTLs) detected are related to albumen quality, Tuiskula-Haavisto et al. (2002) identified regions located on chromosome 2 that affected the Haugh unit. The QTL located on chromosomes 7 and Z are associated with albumen quality at 40 weeks old, while a region located on chromosomes 4 and 26, detected by Honkatukia et al. (2013), could explain 2 %–4 % of phenotypic variance. Far less attention has been paid to longitudinal albumen quality, although it is an age-dependent complex trait (Honkatukia et al., 2005). Wolc et al. (2014) reported that the AH of brown layer hens was affected by different QTL regions at different ages. Consequently, it is necessary to study longitudinal albumen quality.

A remarkable range of discoveries from genome-wide association studies (GWASs) have been made in the past decade (Visscher et al., 2017), both in human and animals. GWAS results have been shown to be useful for prediction and selection for phenotypic traits by a customized gene chip. In the present study, we conducted GWAS analysis on the dynamic albumen quality at 11 time points using a 600 K high-density SNP array in an $F_{2}$ resource population. The main goals of our work were to dissect the genomic loci and genes that contribute to the albumen quality and lay a foundation for future QTL detection of albumen traits in chickens.

## 2 Materials and methods

### 2.1 Population and trait collection

Our experiment was carried out in the Jiangsu Institute of Poultry Science, Yangzhou, China. Standard conditions were maintained throughout and a daily cycle of 16 h light and 8 h dark. A diet corresponding to National Research Council requirements was provided to laying hens, which were fed and watered ad libitum. White Leghorns (WL) and Dongxiang Blue-Shelled (DX) chickens, a Chinese indigenous breed, were crossed to generate the $F_{0}$ population. Six DX males were mated with 80 WL females and 6 WL males were mated with 133 DX females to generate $F_{1}$ populations of 552 and 1029 chicks, respectively. An $F_{2}$ resource population of 1534 chicks was generated from WL/DX (25 males, 407 females) and DX/WL (24 males, 235 females) in a single hatch originating from 49 half-sib and 590 full-sib $F_{1}$ population. More details on the source and housing in the current experiment are described in previous reports (Yuan et al., 2015; Li et al., 2015; Yi et al., 2015; Sun et al., 2015; Shen et al., 2016).

Albumen quality, including AH and HU, was measured in eggs of the first lay from each of the hens and then in eggs every 4 weeks from 32 to 60 weeks of age, and 6 weeks from 60 to 72 weeks of age. Fresh eggs were collected within 1 week. Two eggs per hen were used for analysis and three eggs when hens first start laying. Then the traits were evaluated with an EA-01 egg analyzer (ORKA Food Technology Ltd, Ramat Hasharon, Israel). The device measures HU by the method of Haugh (1937).

Data on descriptive statistics and correlation analysis were calculated with the R v3.0.3 project. The “rntransform” function in the GenABEL package (Aulchenko et al., 2007) of R v3.0.3 was used for the rank-based inverse normal transformations of trait deviations.

### 2.2 Genotyping and quality control 

Nucleic acids were extracted by phenol / chloroform from 1534 blood samples collected from venipuncture. The genotyping data were from a 600 K Affymetrix Axiom Chicken Genotyping Array (Affymetrix, Inc. Santa Clara, CA, USA). The Axiom GT1 algorithm in Affymetrix Power Tools v1.16.0 (APT) software was used for genotype calling and quality control (QC). Sequences with a dish quality control (DQC) ≤0.82 and call rate ≤97 % were excluded from the subsequent analyses. Then, 1534 individuals and 532 299 SNPs remained valid after the application of APT for QC. To enhance the quality of the detection, further QC was carried out using the PLINK v1.90 program (Purcell et al., 2007), with missing rate minor allele frequency (MAF) <5 % and Hardy–Weinberg equilibrium (HWE) $p<1\times 10^{-6}$. We imputed the sporadic missing genotypes by using the BEAGLE v4.0 package (Browning and Browning, 2009); SNPs were retained only if the imputation quality score was $R^{2}>0.5$. Finally, a total of 1512 samples and 435 867 SNPs were used in subsequent analysis. Detailed information on the quality control has been described in previous papers (Yuan et al., 2015; Yi et al., 2015; Sun et al., 2015; Shen et al., 2016).

### 2.3 Genome-wide association analysis

Principal component analysis (PCA) was conducted in the PLINK package to prevent spurious associations that can result from hidden population stratification or cryptic relatedness. Thresholds to determine significant or suggestive genome-wide associations were determined by the “simpleM” method (Gao et al., 2010) with correction for the number of multiple tests. After Bonferroni adjustment, we obtained 59 308 independent results. Hence the significance levels for genome-wide significant and suggestive values were obtained as explained in a previous paper (Shen et al., 2016), which were $8.43\times 10^{-7}$ (0.05/59308) and $1.69\times 10^{-5}$ (1.00/59308), respectively.

The albumen quality at each point was first analyzed using a univariate linear mixed model. After the quality control of genotype data, the univariate analysis was implemented using the GEMMA v0.94 package (Zhou and Stephens, 2014). The significance level was calculated from the P value derived from the Wald test. The univariate linear mixed model is as follows:

1 y=Wα+Xβ+Zμ+ε,

where y is an n×1 vector of phenotypic values for n individuals; **W** is an n×c matrix of covariates (fixed effects, top five principal components (PCs) in our study including a column of ones); α is a c×1 vector of the corresponding coefficients including the intercept; **X** is an n×1 vector of the genotypes of the SNP marker; β is the substitution effects of marker; Z is an n×n relatedness matrix of random effects; μ is an n×1 vector of random effects; and ε is an n×1 vector of errors.

When the SNPs that had suggestive associations with a phenotype at the time points in univariate analysis were included in the subsequent multivariate analysis to avoid computational issues when considering all points simultaneously, the formula is given as follows:

2 $$Y^{*}=\left(W⊗I\right)\alpha^{*}+\left(X⊗I\right)\beta^{*}+\left(Z⊗I\right)\mu^{*}+\varepsilon^{*},$$

where $Y^{*}$ is an n×d matrix of d phenotypes for n samples, W is an n×c matrix of covariates (fixed covariates, top five PCs in our study including a column of ones); $\alpha^{*}$ is a c×d matrix of corresponding coefficients including the intercept; X is an n vector of marker genotypes; and $\beta^{*}$ is a d vector of the substitution effects of marker. Z is an n×d relatedness matrix of random effects, $Z=\left[\begin{array}{ccc} Z_{1} & 0 & 0\\ 0 & Z_{2} & 0\\ 0 & 0 & Z_{d} \end{array}\right]$, $Z_{d}$ are the incidence matrices relating phenotype of the dth trait to random effects; $\mu^{*}$ is an n by d matrix of random effects; $\varepsilon^{*}$ is an n by d matrix of errors; I is the identity matrix.

The Manhattan and Q–Q plots were created by the “gap” and “qqman” packages (Zhao, 2007) in the R project. The GenABEL package (Aulchenko et al., 2007) in the R project was used to calculate the genomic inflation factor resulting from the estimate of false positive signals.

### 2.4 Linkage disequilibrium analysis

We used Haploview v4.2 software (Barrett et al., 2005) to analyze linkage disequilibrium (LD) between significant markers for loci that have a strong linkage to causal mutants. A strong block was defined as a region with LD ($r^{2}\geq 0.33$) between all significant SNPs.

### 2.5 Estimation of variance explained and gene annotation

SNP-based heritability ($h_{\mathrm{snp}}^{2}$ as genomic heritability) from the GWAS was estimated by univariate restricted maximum likelihood in the GCTA v1.24 program (Yang et al., 2011). We used the bivariate mixed model to estimate pairwise phenotypic and genetic correlations simultaneously for each result relating to albumen quality. The genetic relationship matrix partitioned the chicken genome into 28 autosomes and identified two linkage groups by calculating the contribution to phenotypic variance (CPV) for each point.

The genes nearest or harboring significant SNPs associated with albumen quality were chosen as candidate locations. Ensemble and the UCSC (http://genome.ucsc.edu/) Genome Browser (*Gallus gallus* genome v5.0) were used to identify annotated genes located in candidate regions.

## 3 Results

### 3.1 Phenotypic description and genetic parameters

The phenotype for albumen quality is given in Table 1. The AH at the age of first egg (AFE) was largest when the egg weight was lowest (Coorey et al., 2015). The AH and HU at most points showed weak phenotypic correlations except at weeks 60, 66, and 72. Trends observed in AH and HU were nearly unchanged with the age of the hen. Moreover, no significant differences were found in AH or HU at different week time points.

**Table 1:** Phenotypic data of albumen quality.

Age (week)	No.	Albumen quality (mean ± SD)		Phenotypic correlation	Genetic correlation
		AH (mm)	HU		
AFE	1464	4.05±0.8	71.32±6.49	0.918*	0.904
32	1455	4.18±0.73	67.26±6.23	0.955*	0.933
36	1436	4.29±0.95	67.48±8.13	0.928*	0.910
40	1446	4.42±0.76	68.02±6.23	0.948*	0.911
44	1393	4.33±0.87	66.78±8.1	0.953*	0.914
48	1201	4.30±0.88	65.54±8.27	0.950*	0.878
52	1199	4.55±0.85	67.64±7.86	0.915*	0.899
56	1321	4.42±0.98	65.93±8.58	0.908*	0.923
60	1336	4.48±0.92	66.30±8.5	0.953*	0.906
66	1287	4.56±1.03	67.21±9.44	0.943*	0.939
72	1250	4.32±0.95	64.45±9.37	0.944*	0.929

No. is number of samples; AFE is age of first egg; AH is albumen height; HU is Haugh unit.* extremely significant correlation between albumen height and Haugh units at the same week.

The heritability of AH ranged from 0.15 to 0.35, which was higher than HU at each point for the HU derived from egg weight. The highest SNP-based heritability estimates of AH and HU were both found at 32 weeks of age ($h_{\mathrm{snp}}^{2}=0.35$, 0.31, respectively).

The phenotypic correlation and genetic parameters are shown in Tables 2 and 3. The phenotypic correlation coefficient by Pearson correlation and the genetic correlation analysis by bivariate GCTA revealed that both AH and HU at multiple ages are highly and positively interrelated. The correlation coefficient between AH and HU at the same point showed a positive and high value. Moreover, the correlation coefficient indicated that AH or HU showed lower genetic correlations with the traits at other points, compared with those among albumen quality from 32 to 72 weeks of age.

**Table 2:** Phenotypic correlation and genetic parameters of albumen height.

Trait	AFE_AH	AH32	AH36	AH40	AH44	AH48	AH52	AH56	AH60	AH66	AH72
AFE_AH	**0.25**	0.65	0.61	0.58	0.73	0.74	0.58	0.50	0.52	0.53	0.41
AH32	0.28	**0.35**	0.99	0.99	0.85	0.91	0.95	0.74	0.87	0.80	0.79
AH36	0.19	0.43	**0.21**	1.00	1.00	0.87	0.97	0.80	1.00	0.87	0.84
AH40	0.25	0.47	0.36	**0.26**	0.96	0.87	0.93	0.62	0.94	0.87	0.82
AH44	0.22	0.38	0.34	0.38	**0.22**	0.86	1.00	0.84	1.00	0.92	0.76
AH48	0.19	0.36	0.28	0.34	0.34	**0.15**	0.96	0.75	0.90	0.90	0.84
AH52	0.18	0.46	0.34	0.40	0.34	0.34	**0.29**	0.86	1.00	0.88	0.78
AH56	0.15	0.31	0.24	0.30	0.23	0.22	0.34	**0.18**	0.79	0.66	0.85
AH60	0.19	0.40	0.33	0.38	0.33	0.35	0.42	0.36	**0.23**	1.00	0.97
AH66	0.19	0.37	0.27	0.40	0.33	0.32	0.39	0.33	0.48	**0.27**	0.94
AH72	0.14	0.38	0.28	0.36	0.34	0.37	0.38	0.32	0.49	0.54	**0.29**

Genetic correlations (upper triangle) and phenotypic correlations (above diagonal) and with heritability estimates in the diagonal. AFE_AH = first egg albumen height; AH32, AH36, AH40, AH44, AH48, AH52, AH56, AH60, AH66, AH72 = albumen height every 4 weeks from 32 to 60 weeks old, and every 6 weeks from 60 to 72 weeks old.

**Table 3:** Phenotypic correlation and genetic parameters of Haugh units (HU).

Trait	AFE_HU	HU32	HU36	HU40	HU44	HU48	HU52	HU56	HU60	HU66	HU72
AFE_HU${}^{*}$	**0.15**	0.36	0.35	0.31	0.39	0.39	0.24	0.14	0.15	0.12	0.15
HU32	0.11	**0.31**	0.97	0.94	0.79	0.88	0.95	0.60	0.77	0.73	0.71
HU36	0.07	0.37	**0.14**	1.00	1.00	0.85	0.98	0.69	0.99	0.84	0.82
HU40	0.13	0.40	0.32	**0.22**	0.95	0.78	0.86	0.55	0.86	0.76	0.72
HU44	0.09	0.29	0.29	0.32	**0.17**	0.82	0.99	0.79	0.99	0.91	0.71
HU48	0.07	0.29	0.22	0.29	0.29	**0.14**	0.93	0.62	0.86	0.85	0.77
HU52	0.04	0.37	0.26	0.33	0.26	0.29	**0.28**	0.82	0.99	0.81	0.72
HU56	0.04	0.28	0.20	0.26	0.20	0.20	0.31	**0.20**	0.78	0.58	0.85
HU60	0.06	0.33	0.26	0.32	0.27	0.31	0.36	0.37	**0.21**	0.99	0.92
HU66	0.07	0.30	0.25	0.34	0.27	0.27	0.32	0.34	0.44	**0.22**	0.89
HU72	0.02	0.30	0.22	0.29	0.27	0.30	0.31	0.31	0.45	0.51	**0.25**

Genetic correlations (upper triangle) and phenotypic correlations (above diagonal) and with heritability estimates in the diagonal. ${}^{*}$ AFE_HU = first egg albumen height; HU32, HU36, HU40, HU44, HU48, HU52, HU56, HU60, HU66, HU72 = Haugh unit every 4 weeks from 32 to 60 weeks old, and every 6 weeks from 60 to 72 weeks old.

### 3.2 Identifying candidate loci by GWAS

**Figure 1:**
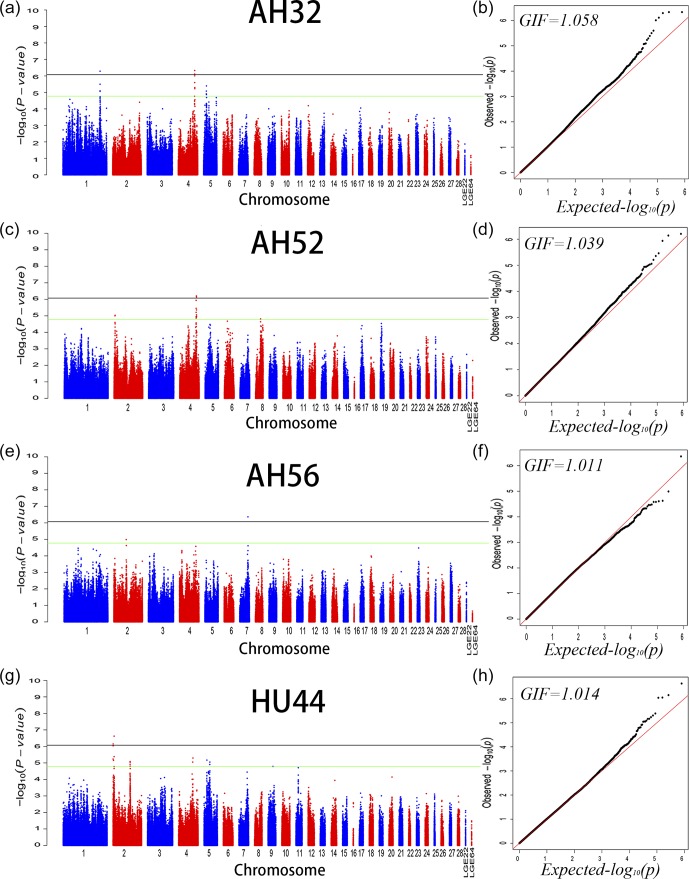
Manhattan plot **(a, c, e, f)** and quantile–quantile (Q–Q) plot **(b, d, f, h)** of the observed P values for albumen height at 32, 52, and 56 weeks of age and Haugh units at 44 weeks of age. For the Manhattan plots, the black and green lines depict the genome-wide significant and suggestive thresholds with values of $8.43\times 10^{-7}$ and $1.69\times 10^{-5}$, respectively.

We performed GWAS analysis for AH and HU at 11 separate time points. Using a univariate method, we found only 3, 2, and 1 SNPs reached a significant level related to AH at 32 (GGA1, GGA4), 52 (GGA4), and 56 (GGA7) week points, respectively. Two SNPs on GGA2 are related to HU at 44 weeks. Moreover, a total of 221 genome-wide suggestive SNPs was obtained from 11 independent univariate analyses, located on 14 (GGA1–9, GGA14, GGA17–20, GGA23, GGA24, GGA30) different chromosomes (Table S1 in the Supplement). From the phenotypic and genetic data obtained above, it is assumed that analysis of the data from 32 weeks of age would be more reliable. A Q–Q and Manhattan plot for all SNPs affecting AH at 32, 52, and 56 weeks and HU at 44 weeks is given in Fig. 1, and the remaining parameters are shown in the Supplement Fig. S1. The detailed significant SNPs by univariate analysis are shown in Table 4.

**Table 4:** SNP markers with significant effects on albumen quality by univariate genome-wide association study.

GGA	SNP	Position	P value	Traits	MAF	EA/AA	Location	Candidate gene	CPV (%)
1	rs14916807	169 300 311	$5.14\times 10^{-7}$	AH32	0.36	A/C	intron	KPNA3	2.48
2	rs314035311	534 249	$7.13\times 10^{-7}$	HU44	0.065	C/T	intron	CDC25A	2.24
2	rs317157401	5 316 830	$2.37\times 10^{-7}$	HU44	0.344	G/C	intron	WDR48	2.29
4	rs315201454	76 457 427	$4.69\times 10^{-7}$	AH32	0.059	G/A	intron	NCAPG	2.00
4	rs313185009	77 654 701	$6.27\times 10^{-7}$	AH52	0.058	G/A	intergenic	FGFBP1	2.47
4	rs313154528	77 924 330	$7.29\times 10^{-7}$	AH52	0.061	G/T	intergenic	BST1	2.42
7	rs312465596	31 130 005	$4.36\times 10^{-7}$	AH56	0.179	C/T	intron	THSD7B	2.39

Abbreviations: GGA, *Gallus gallus * chromosome; EA, effect allele (minor allele); AA, alternative allele (major allele); MAF, minor allele frequency; CPV, contribution to phenotypic variance (%).

**Figure 2:**
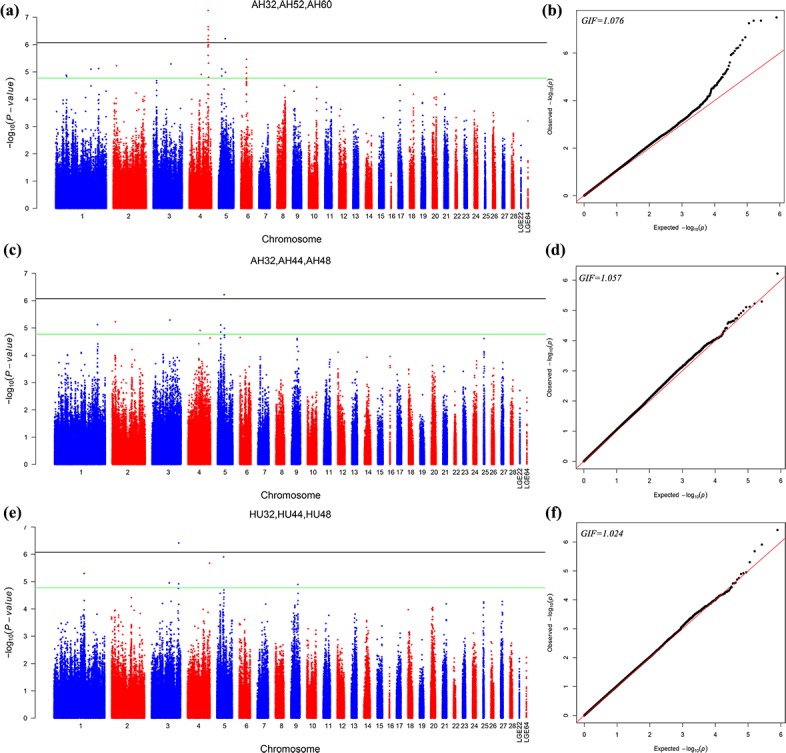
Manhattan plot **(a, c, e)** and quantile–quantile (Q–Q) plot **(b, d, f)** of the observed P values for the albumen quality by multivariate analysis. For the Manhattan plots, the black line and green lines are the genome-wide significant and suggestive thresholds with values of $8.43\times 10^{-7}$ and $1.69\times 10^{-5}$, respectively.

The different points that had similar significant or suggestive SNP regions were performed by a multivariate model. Then we conducted GWAS for the AH and HU at 32, 44, and 48 weeks of age. Moreover, the AH at 32, 52, and 60 weeks were also conducted by multivariate analysis in GEMMA. Consequently, nine loci exceeded the threshold for genome-wide significance association with AH at 32, 52, and 60 weeks. Figure 2 depicts the Manhattan and Q–Q plots for all SNPs. Detailed information about significant and suggestive SNPs by multivariate analysis are shown in Table S2.

One locus on GGA5 and nine loci on GGA4 provided convincing evidence for associations with AH at week 32. In addition, one locus on GGA3 was related to HU at 32, 44, and 48 weeks old. The results are given in Table 5.

**Table 5:** Eleven SNP markers with significant effects on albumen quality by multivariate analysis.

GGA	SNP	Position	P value	Traits	MAF	EA/AA	Location	Candidate gene
4	rs14491074	76 631 420	$3.21\times 10^{-8}$	AH32, AH52, AH60	0.054	C/T	intergenic	LDB2
4	rs315201454	76 457 427	$4.38\times 10^{-8}$	AH32, AH52, AH60	0.057	G/A	intron	NCAPG
4	rs316243629	76 467 271	$4.38\times 10^{-8}$	AH32, AH52, AH60	0.057	A/G	intron	NCAPG
4	rs14491030	76 458 342	$5.63\times 10^{-8}$	AH32, AH52, AH60	0.058	G/A	missense	NCAPG
4	rs314745738	76 406 489	$2.19\times 10^{-7}$	AH32, AH52, AH60	0.227	G/A	intron	LCORL
4	rs16437368	77 205 038	$2.86\times 10^{-7}$	AH32, AH52, AH60	0.051	C/T	intron	BST1
4	rs313185009	77 654 701	$4.71\times 10^{-7}$	AH32, AH52, AH60	0.059	G/A	intergenic	FGFBP1
4	rs314487178	76 400 165	$6.44\times 10^{-7}$	AH32, AH52, AH60	0.227	T/C	intron	LCORL
4	rs15619270	76 450 114	$8.18\times 10^{-7}$	AH32, AH52, AH60	0.056	C/G	intergenic	NCAPG
5	rs314039089	28 424 606	$6.06\times 10^{-7}$	AH32, AH44, AH48	0.280	T/C	intergenic	RAD51B
3	rs314618917	104 795 435	$3.87\times 10^{-7}$	HU32, HU44, HU48	0.083	A/G	intron	ZNF512

Abbreviations: SNP, single nucleotide polymorphism; GGA, *Gallus gallus * chromosome; EA, effect allele (minor allele); AA, alternative allele (major allele); MAF, minor allele frequency.

### 3.3 SNP effects on albumen quality 

**Figure 3:**
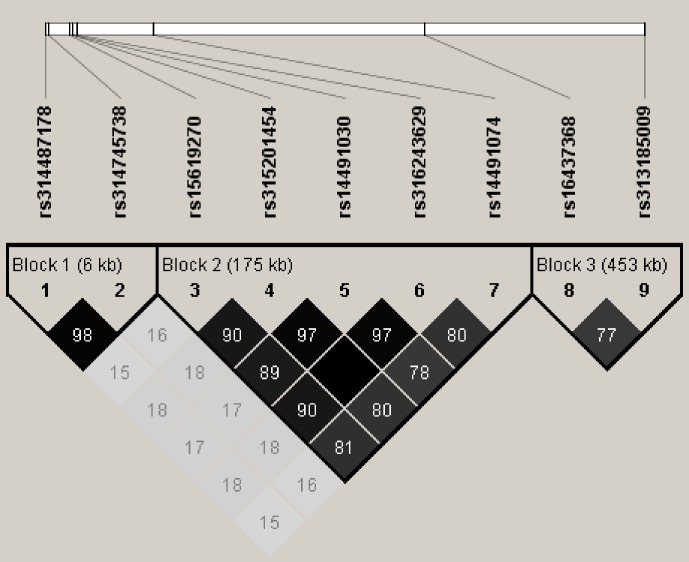
LD analysis of the significant SNPs on GGA4. Three strong blocks were observed in this significant region, number in the diamond block is the $R^{2}$ value.

LD analysis of the significant SNPs on GGA4 showed that there are three strong LD blocks (Fig. 3) in the DX and WL crossed population, corresponding with the SNPs *rs314487178*, *rs14491030*, and *rs313185009*. Overall, the SNPs obtained from univariate analysis explained more than 2 % of the phenotypic variance for the AH. The proportion of SNPs obtained from multivariate analysis was highest at 32, 44, 52, and 56 weeks old. To detect candidate genes, the significant SNPs were used to blast the *Gallus gallus* assembly 5.0 on Ensemble. The detailed genes are showed in Tables 4 and 5. It is noteworthy that SNP*rs14491030 * located in the missense area of gene *NCAPG* has a significant association with AH. The largest estimate was obtained from AH at 52 weeks old with the proportion reaching 2.47 %. The CPV of the remaining SNPs ranged from 0 % to ∼2 % (Table 6).

**Table 6:** Contributions to the phenotypic variance in albumen quality by eight mutations at different weeks.

GGA	SNP		CPV (%)										
		Trait/age	AFE	32	36	40	44	48	52	56	60	66	72
1	rs14916807	AH	0.319	2.477	1.440	0.628	0.875	1.679	0.001	0.788	0.304	0.356	0.131
2	rs314035311	HU	0.001	0.001	0.001	0.057	2.245	0.262	0.001	0.001	0.001	0.001	0.001
2	rs317157401	HU	0.001	0.134	0.449	0.605	2.292	0.433	0.421	0.319	0.438	0.407	0.267
3	rs314618917	HU	0.001	0.926	0.460	0.383	0.290	0.743	0.591	0.001	0.662	0.745	0.549
4	rs14491030	AH	0.034	1.836	0.677	0.705	0.736	0.706	1.964	1.293	0.556	0.042	0.375
4	rs313185009	AH	0.047	1.637	0.360	0.413	0.566	0.802	1.635	1.022	0.368	0.098	0.583
5	rs314039089	AH	0.001	0.001	0.465	0.370	2.333	0.001	0.147	0.001	0.139	0.464	0.349
7	rs312465596	AH	0.001	0.126	0.471	0.201	0.710	0.001	0.001	2.393	0.245	0.897	0.001

Abbreviations: CPV, Contributions to the phenotypic variance; AH, albumen height; HU, Haugh unit; AFE, age at first egg.

## 4 Discussion

Previous genome-wide studies have focused on albumen quality from a limited time point, with different results from different reports. In the current research, we integrated multiple ages, a high-density array, and statistical analysis strategies with an aim to understand the molecular mechanisms of albumen quality in chickens.

The value of AH ranged from 4.05 to 4.56 mm when measured by the egg analyzer, which was lower than the conventional method used in previous reports (Ledur et al., 2002; Sert et al., 2011; Honkatukia et al., 2013; Goto et al., 2014; Rath et al., 2015). The first reason for this is that the egg analyzer is an automatic testing device, therefore the albumen at the thickest point is not within the range of testing because the testing area is in a relatively fixed position. It is assumed that the conventional method may be a more reliable method to test AH. Second, the AH of unselected local hens had a lower albumen compared to commercial hens (Kehua et al., 2012). Our population of $F_{0}$ is generated from a local Chinese chicken, the DX chicken, which has a lower AH than commercial lines.

Heritability estimation on AH and HU by GCTA was lower, except for the value at 32 weeks of age, when compared to previous studies (Honkatukia et al., 2013; Goto et al., 2014; Rath et al., 2015; Wolc et al., 2012). The genetic correlation between AH at different time points was from 0.41 to 1.0, while the phenotypic correlation was lower. The genetic parameters estimated from genomic relationship will be more accurate compared to pedigree relationship (Wolc et al., 2013), and are easily affected by the structure of statistical models, depth of pedigree, and data size. On the other hand, this suggests that the trait of albumen quality is easily affected by the environment; moreover, given that high-density SNP genotypes increased accuracies of estimated breeding value (Wolc et al., 2013), it is likely that genomic selection should be applied in albumen quality breeding.

We obtained a total of seven significant SNPs located on GGA1, 2, 4, and 7 that were associated with AH and HU by univariate analysis, while 10 SNPs were found mainly on GGA4 by conducting multivariate analysis, which indicated that common variants were important contributors to the albumen quality. The HU is influenced by the strain and age, the calculation of which has been questioned (Silversides and Villeneuve, 1994). In our results, the significant number of SNPs that were associated with HU was less than for AH, this may be because of the overcompensation for egg weight in the HU formula (Silversides, 1994; Nestor and Jaap, 1963). It suggests that only taking AH into account to describe albumen quality would be sufficient.

The QTL affecting albumen quality was on different chromosomes with different breeds in previous reports. Goto et al. (2014) found the region associated with albumen was located on GGA1, 2, 4, 5, 6, 8, 9, 27, and Z. Honkatukia et al. (2013). obtained QTL on chromosomes 7 and Z chromosome related to HU, and Wolc et al. (2014) reported that a region on GGA2 significantly affected AH. Combined with the results from our previous study, this suggests that albumen quality is a complex quantitative trait affected by polygenes.

From our study, evidence for the region between position 76.4 to 77.7 Mb on GGA4 associated with many traits corresponded to the previous findings of different resource populations (Schreiweis et al., 2006; Kerje et al., 2003), which suggests that GGA4 may play a key role in different traits. Moreover, our study identified candidate genes on GGA4 that provide strong confirmation of our previously reported region for egg weight (Yi et al., 2015) and eggshell traits (Sun et al., 2015). It is noted that the QTL that affects egg weight or shell traits also influences albumen quality. The significant SNPs on GGA4 were distributed into two blocks. The *rs14491030* located in the missense of gene *NCAPG* (non-SMC condensin I complex, subunit G). The gene has a pleiotropy function on many traits, like residual feed intake in bovines (Widmann et al., 2015), withers height in horses (Tetens et al., 2013), and body weight in chickens (Setoguchi et al., 2009). The results of our population study revealed that *NCAPG* may affect egg weight because albumen is the major contributor to egg weight (Silversides and Budgell, 2004). The egg weight gain may be through increasing albumen by *NCAPG* modulation. Another SNP *rs313185009 *located on *FGFBP1* (fibroblast growth factor, FGF, binding protein 1) showed 1.637 % CPV for AH at 32 weeks.* FGFBP1* binds to FGFs to play a role in the control of wound healing and tumor angiogenesis in humans (Tomaszewski et al., 2011). In chickens, the release of FGFs from local extracellular matrix storage is essential for chicken embryonic development within the first 3 days (Gibby et al., 2009). Many genes displayed a pleiotropic effect in multiple phenotypes simultaneously (Mackay et al., 2009); it is speculated that *FGFBP1 *may be involved in albumen synthesis.

On GGA1, two regions around 50 Mb (Tuiskula-Haavisto et al., 2004) and between 90.35 and 123.03 Mb (Yi, 2005) are associated with albumen quality. The locus *rs14916807* located on GGA1 with a physical position of 169.3 Mb was significantly associated with AH at 32 weeks old, and the CPV (2.48 %) was highest in all significant loci. Findings obtained from other reports showed that this region was also related to body weight (Wang et al., 2015; Xie et al., 2012) and bone traits (Zhang et al., 2010), which indicated that the region has a pleiotropic function on many traits. The locus is located in the intron of gene* KPNA3 *(karyopherin alpha 3).* KPNA3* affects breast muscle weight and leg muscle weight in chicken (Xie et al., 2012), and is highly expressed in adipose tissue acting on the importation of proteins in rat (Plant et al., 2006). The changes in ovomucin significantly affect thick albumen (Toussant and Latshaw, 1999). The aforementioned genes may participate in the process of synthesis and transportation of ovomucin.

Notably, it was shown that the genomic region on 24.52–29.72 Mb overlapped on GGA7 based on *galgal 4.0* association with AH and HU at the age of 40 weeks (Honkatukia et al., 2013). Our results revealed that the* rs312465596* at position 31.13 Mb based on *galgal 5.0* also influences albumen height at 56 weeks old. Accordingly, it was revealed that the region around 31 Mb on GGA7 may be a candidate QTL for albumen quality. The SNP *rs312465596 *located in gene *THSD7B* (thrombospondin type-1 domain-containing protein 7B precursor). *THSD7B* affects atherothrombotic disease in humans (Brand-Herrmann, 2008). Further study on how this gene affects albumen quality is necessary.

Two adjacent SNPs on GGA2 in our present study were significantly associated with HU at 44 weeks, which did not coincide with previous reports that show a QTL located at 10.05 Mb related to AH at 40 weeks (Liu et al., 2011). The nearest genes to the two markers were* CDC25A *and* WDR48*. The gene* CDC25A* (cell division cycle 25A) is a crucial regulator of cell cycle progression (Shreeram et al., 2008), while* WDR48* (WD repeat protein 48) is considered a potential tumor suppressor (Gangula and Maddika, 2013), yet its function on albumen quality is almost unknown. Moreover, another two SNPs on GGA5 and GGA3 association with AH and HU at three different ages did not agree with a previously reported region on the same chromosome (Abasht et al., 2009). The results show compellingly that albumen height is a complex trait that is affected by polygenes or multiple QTL regions.

Although the results from the current study may be more vulnerable to different regions at longitudinal points, the present research strongly suggests that at least one major QTL on GGA4 and several other loci of minor effects are involved in albumen quality, which suggests that the genetic architecture of albumen quality is partially discrete at different QTL regions and these QTLs have age-dependent manners of controlling albumen trait (Goto et al., 2018). This has important ramifications for understanding complex trait interactions and pleiotropy in domestication. A complex trait is often a quantitative trait that is affected by polygenes. Moreover, most of the genes detected in the current research have not been reported in association with egg quality in previous work. This highlights that pleiotropy may occur between traits that are not thought to be functionally related (Mackay et al., 2009). For example, some diseases in humans are not physiologically associated but can still be affected by the same mutation (Flint and Mackay, 2009). Albumen quality is a complex trait that involves transportation, protein synthesis, and secretion; it is assumed that genetic relationships exist between the albumen and other traits in the process of albumen formation. Despite all this, the impact on albumen quality of the genes identified by us requires further investigation.

## 5 Conclusions

From the current research, one major QTL detected on GGA4 showed significant association with albumen quality. In this region, *NCAPG* and *FGFBP1* were analyzed as candidate genes. Moreover, it was found that putative QTLs at GGA1, GGA2, GGA7, GGA5, and GGA3 were associated with albumen quality. This result suggests that albumen quality is a complex trait that is affected by one major QTL and polygenes.

## Supplement

10.5194/aab-62-113-2019-supplement The supplement related to this article is available online at: https://doi.org/10.5194/aab-62-113-2019-supplement.

## Data Availability

The datasets used and/or analyzed during the current research are available upon request.

## References

[bib1.bib1] Abasht B, Sandford E, Arango J, Settar P, Fulton JE, O'Sullivan NP, Hassen A, Habier D, Fernando RL, Dekkers JC, Lamont SJ (2009). Extent and consistency of linkage disequilibrium and identification of DNA markers for production and egg quality traits in commercial layer chicken populations. BMC Genomics.

[bib1.bib2] Abeyrathne ED, Lee HY, Ahn DU (2013). Egg white proteins and their potential use in food processing or as nutraceutical and pharmaceutical agents–a review. Poultry Sci.

[bib1.bib3] Aulchenko YS, Ripke S, Isaacs A, Van Duijn CM (2007). GenABEL: an R library for genome-wide association analysis. Bioinformatics (Oxford, England).

[bib1.bib4] Barrett JC, Fry B, Maller J, Daly MJ (2005). Haploview: analysis and visualization of LD and haplotype maps. Bioinformatics (Oxford, England).

[bib1.bib5] Brand-Herrmann SM (2008). Where do we go for atherothrombotic disease genetics?. Stroke.

[bib1.bib6] Browning BL, Browning SR (2009). A unified approach to genotype imputation and haplotype-phase inference for large data sets of trios and unrelated individuals. Am J Hum Genet.

[bib1.bib7] Campbell L, Raikos V, Euston SR (2003). Modification of functional properties of egg-white proteins. Die Nahrung.

[bib1.bib8] Coorey R, Novinda A, Williams H, Jayasena V (2015). Omega-3 fatty acid profile of eggs from laying hens fed diets supplemented with chia, fish oil, and flaxseed. J Food Sci.

[bib1.bib9] Flint J, Mackay TF (2009). Genetic architecture of quantitative traits in mice, flies, and humans. Genome Res.

[bib1.bib10] Gangula NR, Maddika S (2013). WD repeat protein WDR48 in complex with deubiquitinase USP12 suppresses Akt-dependent cell survival signaling by stabilizing PH domain leucine-rich repeat protein phosphatase 1 (PHLPP1). J Biol Chem.

[bib1.bib11] Gao X, Becker LC, Becker DM, Starmer JD, Province MA (2010). Avoiding the high Bonferroni penalty in genome – wide association studies. Genet Epidemiol.

[bib1.bib12] Gibby KA, McDonnell K, Schmidt MO, Wellstein A (2009). A distinct role for secreted fibroblast growth factor-binding proteins in development. P Natl Acad Sci USA.

[bib1.bib13] Goto T, Ishikawa A, Yoshida M, Goto N, Umino T, Nishibori M, Tsudzuki M (2014). Mapping of Main-Effect and Epistatic Quantitative Trait Loci for Internal Egg Traits in an F2 Resource Population of Chickens. J Poultry Sci.

[bib1.bib14] Goto T, Ishikawa A, Nishibori M, Tsudzuki M (2018). A longitudinal quantitative trait locus mapping of chicken growth traits. Mol Genet Genomics.

[bib1.bib15] Haugh R (1937). The Haugh unit for measuring egg quality. United States Egg and Poultry Magazine.

[bib1.bib16] Honkatukia M, Tuiskula-Haavisto M, de Koning DJ, Virta A, Maki-Tanila A, Vilkki J (2005). A region on chicken chromosome 2 affects both egg white thinning and egg weight. Genet Sel Evol.

[bib1.bib17] Honkatukia M, Tuiskula-Haavisto M, Arango J, Tabell J, Schmutz M, Preisinger R, Vilkki J (2013). QTL mapping of egg albumen quality in egg layers. Genetics, selection, evolution, Genet Sel Evol.

[bib1.bib18] Kehua W, Taocun D, Liang Q, Jun G, Jun H (2012). Comparison and Analysis for Egg Quality of Seven Breeds of Layer. China Poultry.

[bib1.bib19] Kerje S, Carlborg O, Jacobsson L, Schutz K, Hartmann C, Jensen P, Andersson L (2003). The twofold difference in adult size between the red junglefowl and White Leghorn chickens is largely explained by a limited number of QTLs. Anim Genet.

[bib1.bib20] Ledur MC, Liljedahl LE, McMillan I, Asselstine L, Fairfull RW (2002). Genetic effects of aging on egg quality traits in the first laying cycle of White Leghorn strains and strain crosses. Poultry Sci.

[bib1.bib21] Li-Chan EC, Kim H-O (2008). Structure and chemical composition of eggs. Egg Bioscience and Biotechnology.

[bib1.bib22] Li J, Yang S, Su N, Wang Y, Yu J, Qiu H, He X (2015). Overexpression of long non-coding RNA HOTAIR leads to chemoresistance by activating the Wnt/
β
-catenin pathway in human ovarian cancer. Tumor Biol.

[bib1.bib23] Liu W, Li D, Liu J, Chen S, Qu L, Zheng J, Xu G, Yang N (2011). A genome-wide SNP scan reveals novel loci for egg production and quality traits in white leghorn and brown-egg dwarf layers. PloS one.

[bib1.bib24] Mackay TF, Stone EA, Ayroles JF (2009). The genetics of quantitative traits: challenges and prospects. Nat Rev Genet.

[bib1.bib25] Nestor KE, Jaap RG (1963). Egg Weight May Influence Albumen Height. Poultry Sci.

[bib1.bib26] Plant KE, Everett DM, Gordon Gibson G, Lyon J, Plant NJ (2006). Transcriptomic and phylogenetic analysis of Kpna genes: a family of nuclear import factors modulated in xenobiotic-mediated liver growth. Pharmacogenet Genomics.

[bib1.bib27] Purcell S, Neale B, Todd-Brown K, Thomas L, Ferreira MA, Bender D, Maller J, Sklar P, De Bakker PI, Daly MJ (2007). PLINK: a tool set for whole-genome association and population-based linkage analyses. Am J Hum Genet.

[bib1.bib28] Rath PK, Mishra PK, Mallick BK, Behura NC (2015). Evaluation of different egg quality traits and interpretation of their mode of inheritance in White Leghorns. Vet World.

[bib1.bib29] Roberts JR (2004). Factors affecting egg internal quality and egg shell quality in laying hens. J Poultry Sci.

[bib1.bib30] Schreiweis M, Hester P, Settar P, Moody D (2006). Identification of quantitative trait loci associated with egg quality, egg production, and body weight in an F2 resource population of chickens1. Anim Genet.

[bib1.bib31] Scott TA, Silversides FG (2000). The effect of storage and strain of hen on egg quality. Poultry Sci.

[bib1.bib32] Sert D, Aygun A, Demir MK (2011). Effects of ultrasonic treatment and storage temperature on egg quality. Poultry Sci.

[bib1.bib33] Setoguchi K, Furuta M, Hirano T, Nagao T, Watanabe T, Sugimoto Y, Takasuga A (2009). Cross-breed comparisons identified a critical 591-kb region for bovine carcass weight QTL (CW-2) on chromosome 6 and the Ile-442-Met substitution in NCAPG as a positional candidate. BMC Genet.

[bib1.bib34] Shen M, Qu L, Ma M, Dou T, Lu J, Guo J, Hu Y, Yi G, Yuan J, Sun C, Wang K, Yang N (2016). Genome-Wide Association Studies for Comb Traits in Chickens. PloS one.

[bib1.bib35] Shreeram S, Hee WK, Bulavin DV (2008). Cdc25A serine 123 phosphorylation couples centrosome duplication with DNA replication and regulates tumorigenesis. Mol Cell Biol.

[bib1.bib36] Silversides FG (1994). The haugh unit correction for egg weight is not adequate for comparing eggs from chickens of different lines and ages. J Appl Poultry Res.

[bib1.bib37] Silversides FG, Budgell K (2004). The relationships among measures of egg albumen height, pH, and whipping volume. Poultry Sci.

[bib1.bib38] Silversides FG, Villeneuve P (1994). Is the Haugh Unit Correction for Egg Weight Valid for Eggs Stored at Room Temperature?. Poultry Sci.

[bib1.bib39] Sun C, Qu L, Yi G, Yuan J, Duan Z, Shen M, Qu L, Xu G, Wang K, Yang N (2015). Genome-wide association study revealed a promising region and candidate genes for eggshell quality in an F2 resource population. BMC Genomics.

[bib1.bib40] Sun C, Liu J, Li W, Xu G, Yang N (2017). Divergent Proteome Patterns of Egg Albumen from Domestic Chicken, Duck, Goose, Turkey, Quail and Pigeon. Proteomics.

[bib1.bib41] Tetens J, Widmann P, Kuhn C, Thaller G (2013). A genome-wide association study indicates LCORL/NCAPG as a candidate locus for withers height in German Warmblood horses. Anim Genet.

[bib1.bib42] Tomaszewski M, Charchar FJ, Nelson CP, Barnes T, Denniff M, Kaiser M, Debiec R, Christofidou P, Rafelt S, van der Harst P, Wang WY, Maric C, Zukowska-Szczechowska E, Samani NJ (2011). Pathway analysis shows association between FGFBP1 and hypertension. J Am Soc Nephrol.

[bib1.bib43] Toussant MJ, Latshaw JD (1999). Ovomucin content and composition in chicken eggs with different interior quality. J Sci Food Agri.

[bib1.bib44] Tuiskula-Haavisto M, Honkatukia M, Vilkki J, de Koning D-J, Schulman NF, Maki-Tanila A (2002). Mapping of quantitative trait loci affecting quality and production traits in egg layers. Poultry Sci.

[bib1.bib45] Tuiskula-Haavisto M, de Koning DJ, Honkatukia M, Schulman NF, Maki-Tanila A, Vilkki J (2004). Quantitative trait loci with parent-of-origin effects in chicken. Genet Res.

[bib1.bib46] Visscher PM, Wray NR, Zhang Q, Sklar P, McCarthy MI, Brown MA, Yang J (2017). 10 Years of GWAS Discovery: Biology, Function, and Translation. Am J Hum Genet.

[bib1.bib47] Wang W, Wang J, Zhang T, Wang Y, Zhang Y, Han K (2015). Genome – wide association study of growth traits in Jinghai Yellow chicken hens using SLAF – seq technology. Animal Genet.

[bib1.bib48] Widmann P, Reverter A, Weikard R, Suhre K, Hammon HM, Albrecht E, Kuehn C (2015). Systems biology analysis merging phenotype, metabolomic and genomic data identifies Non-SMC Condensin I Complex, Subunit G (NCAPG) and cellular maintenance processes as major contributors to genetic variability in bovine feed efficiency. PloS one.

[bib1.bib49] Wolc A, Arango J, Settar P, O'Sullivan NP, Olori VE, White IM, Hill WG, Dekkers JC (2012). Genetic parameters of egg defects and egg quality in layer chickens. Poultry Sci.

[bib1.bib50] Wolc A, Arango J, Jankowski T, Settar P, Fulton JE, O'Sullivan NP, Fernando R, Garrickf DJ, Dekkers JC (2013). Pedigree and genomic analyses of feed consumption and residual feed intake in laying hens. Poultry Sci.

[bib1.bib51] Wolc A, Arango J, Jankowski T, Dunn I, Settar P, Fulton J, O'Sullivan N, Preisinger R, Fernando R, Garrick D (2014). Genome – wide association study for egg production and quality in layer chickens. J Anim Breeding Genetics.

[bib1.bib52] Xie L, Luo C, Zhang C, Zhang R, Tang J, Nie Q, Ma L, Hu X, Li N, Da Y (2012). Genome-wide association study identified a narrow chromosome 1 region associated with chicken growth traits. PloS one.

[bib1.bib53] Yang J, Lee S, Goddard M, Visscher P (2011). GCTA: a tool for genome-wide complex trait analysis. Am J Hum Genet.

[bib1.bib54] Yi G, Shen M, Yuan J, Sun C, Duan Z, Qu L, Dou T, Ma M, Lu J, Guo J, Chen S, Qu L, Wang K, Yang N (2015). Genome-wide association study dissects genetic architecture underlying longitudinal egg weights in chickens. BMC Genomics.

[bib1.bib55] Yi N (2005). Identification of QTL for production traits in chickens. Anim Biotechnol.

[bib1.bib56] Yuan J, Wang K, Yi G, Ma M, Dou T, Sun C, Qu L-J, Shen M, Qu L, Yang N (2015). Genome-wide association studies for feed intake and efficiency in two laying periods of chickens. Genet Sel Evol.

[bib1.bib57] Zhang H, Zhang YD, Wang SZ, Liu XF, Zhang Q, Tang ZQ, Li H (2010). Detection and fine mapping of quantitative trait loci for bone traits on chicken chromosome one. J Anim Breed & Genet.

[bib1.bib58] Zhao JH (2007). gap: Genetic Analysis Package. J Stat Softw.

[bib1.bib59] Zhou X, Stephens M (2014). Efficient multivariate linear mixed model algorithms for genome-wide association studies. Nature Methods.

