# Supplementary material for: Identification of potential genomic regions and candidate genes for egg albumen quality by a genome-wide association study
Source: Arch Anim Breed. 2019 Mar 25;62(1):113–23. doi: 10.5194/aab-62-113-2019 (PMC6853030; doi:10.5194/aab-62-113-2019)
Supplement: The supplement related to this article is available online at: https://doi.org/10.5194/aab-62-113-2019-supplement. [file aab-62-113-supplement.zip › aab-62-113-2019-supplement-title-page.pdf]

Supplement of Arch. Anim. Breed., 62, 113–123, 2019
https://doi.org/10.5194/aab-62-113-2019-supplement
© Author(s) 2019. This work is distributed under
the Creative Commons Attribution 4.0 License.

Open Access
Archives Animal Breeding

*Supplement of*

# Identification of potential genomic regions and candidate genes for egg albumen quality by a genome-wide association study

**Liang Qu et al.**

*Correspondence to:* Kehua Wang (sqbreeding@126.com) and Honglin Liu (liuhonglin@njau.edu.cn)

- aab-62-113-2019-supplement-title-page.pdf
- Additional file 1.xlsx
- Fig S1.pdf

The copyright of individual parts of the supplement might differ from the CC BY 4.0 License.
