# Supplementary figures and images for: Identification of potential genomic regions and candidate genes for egg albumen quality by a genome-wide association study
Source: Arch Anim Breed. 2019 Mar 25;62(1):113–23. doi: 10.5194/aab-62-113-2019 (PMC6853030; doi:10.5194/aab-62-113-2019)

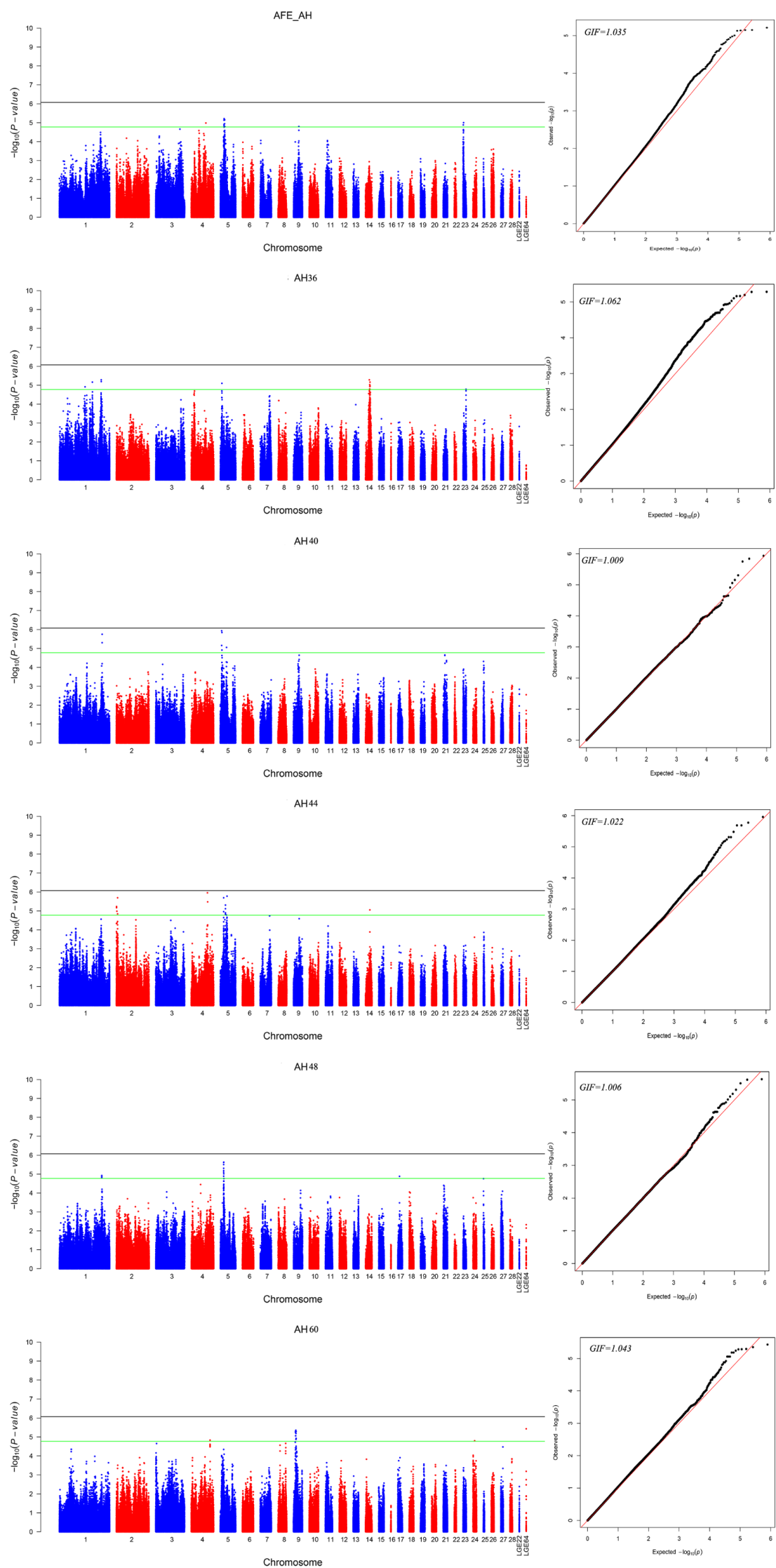

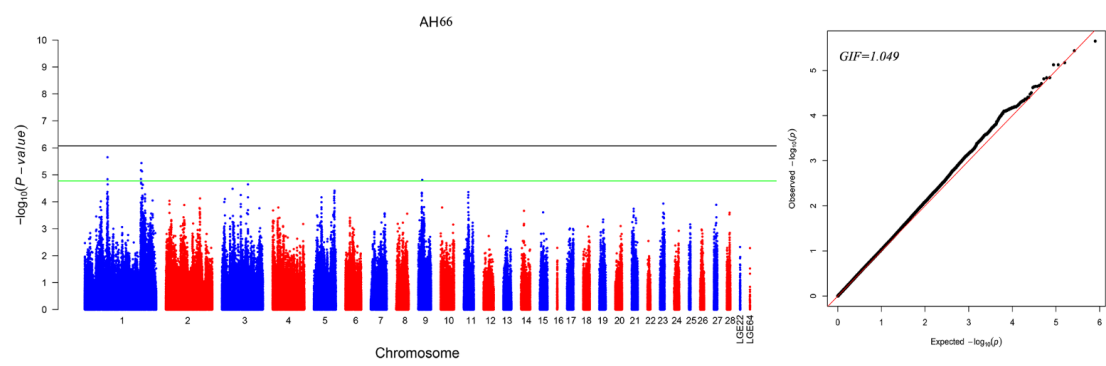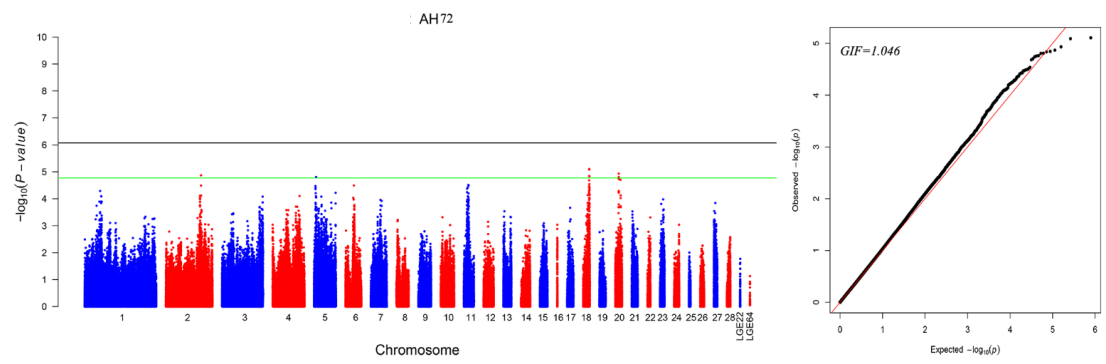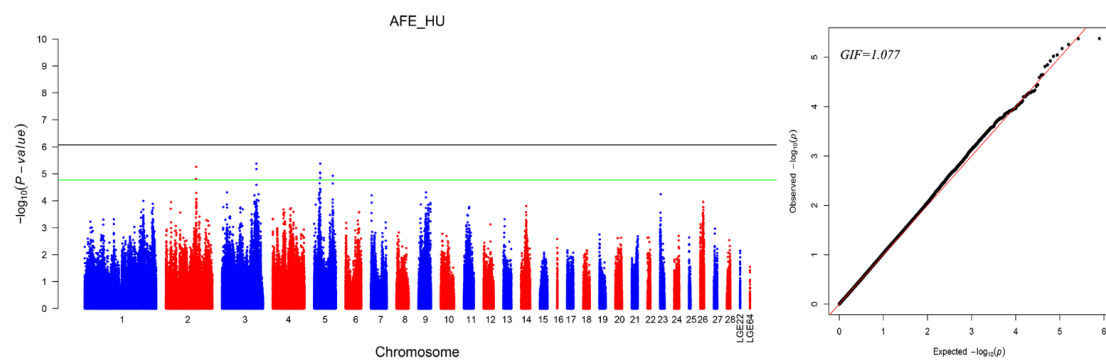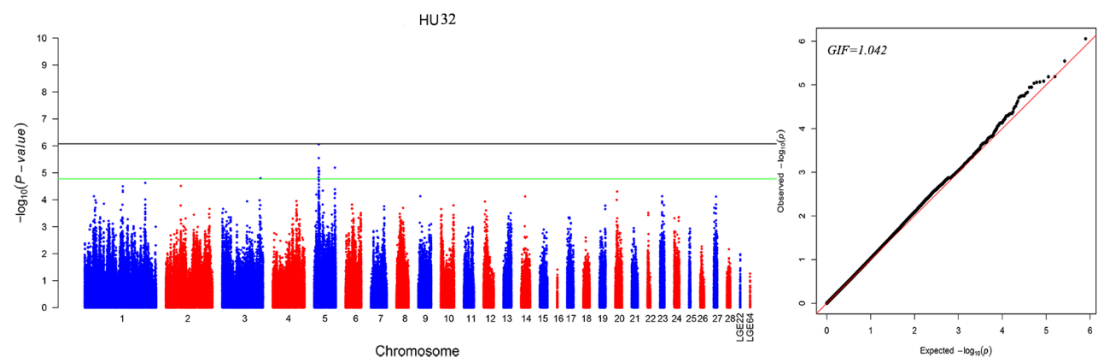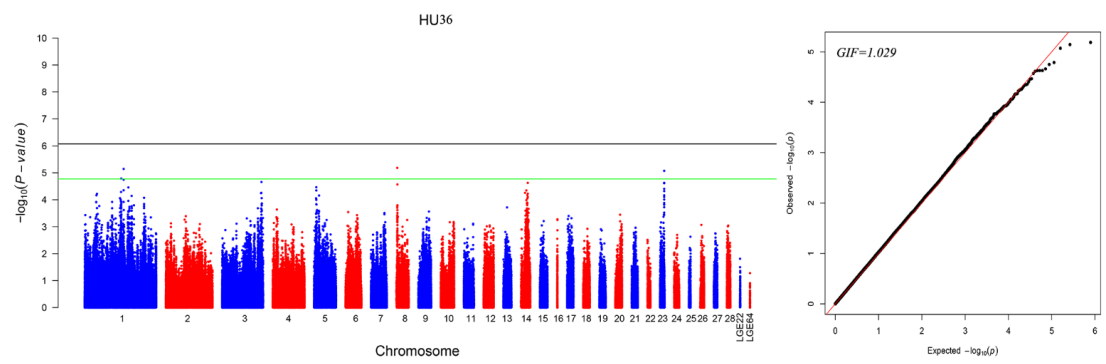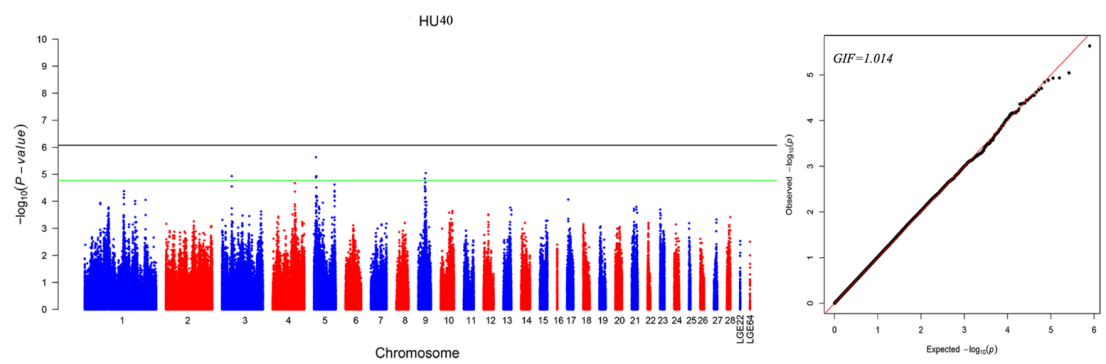

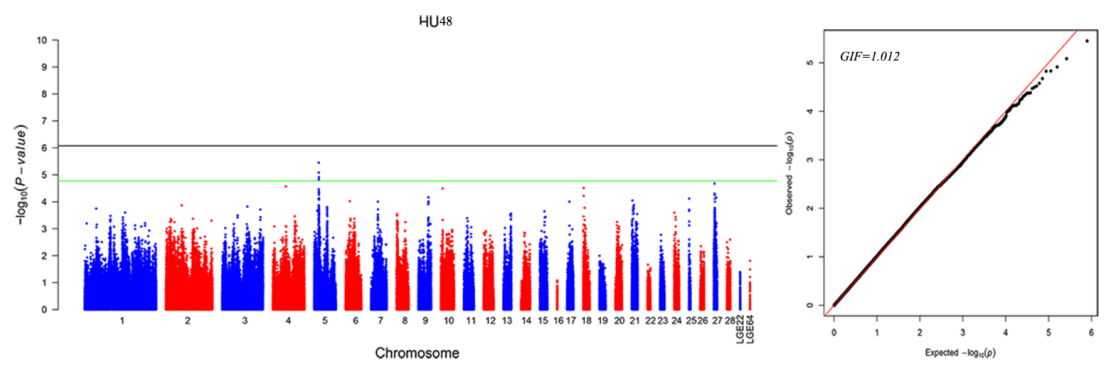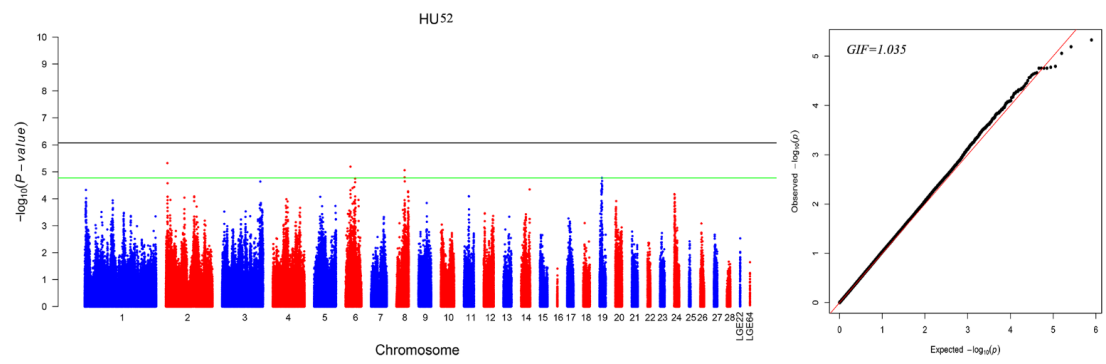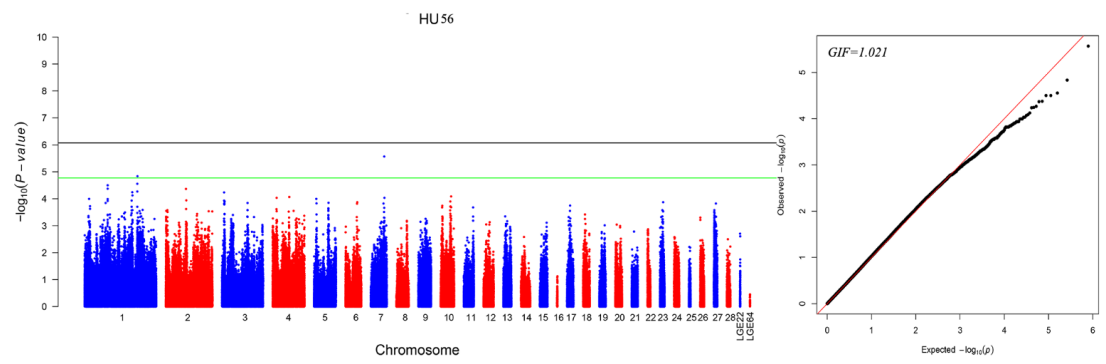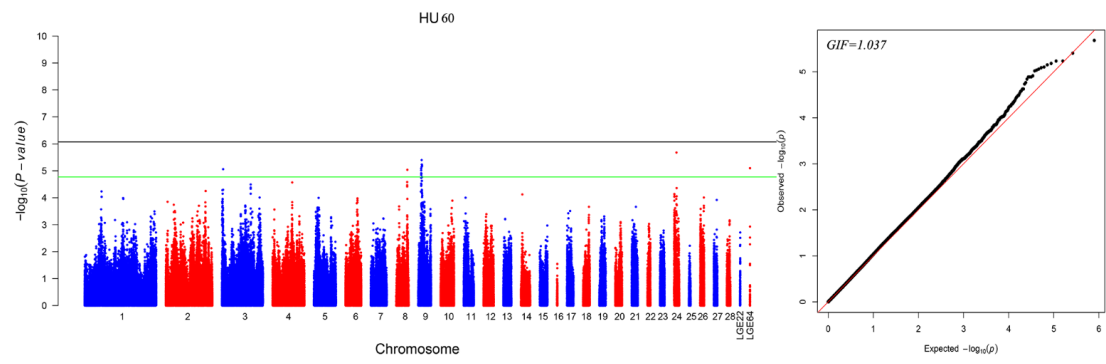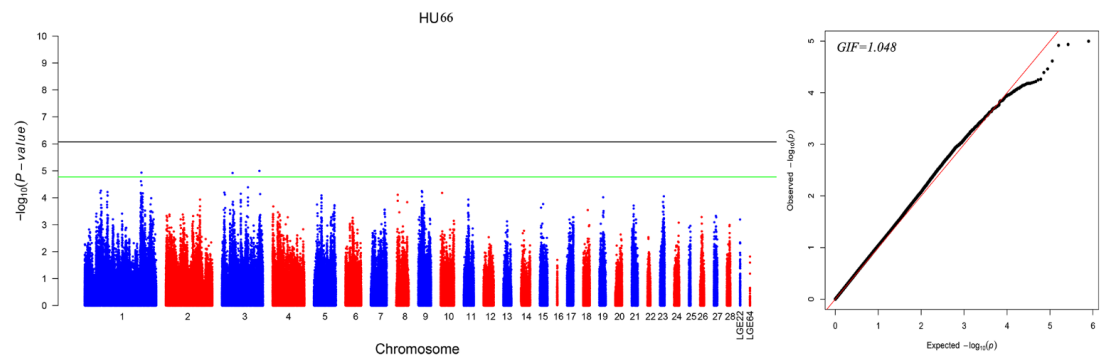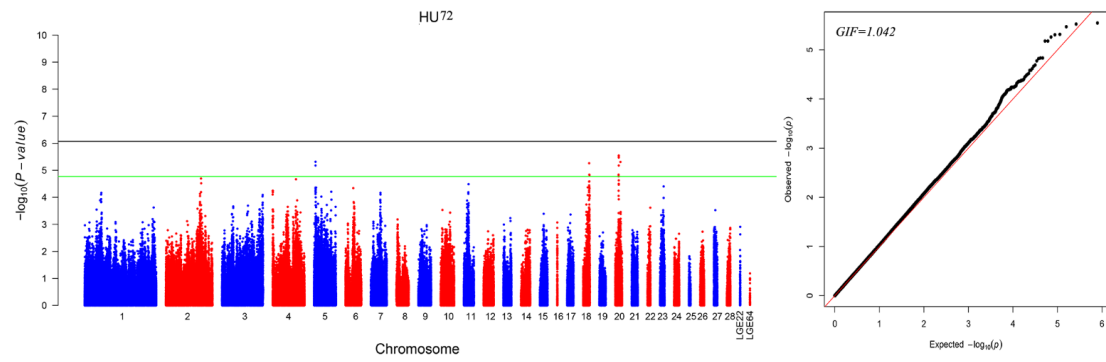

Supplement: The supplement related to this article is available online at: https://doi.org/10.5194/aab-62-113-2019-supplement. [file aab-62-113-supplement.zip › Fig S1.pdf]
